# Context dependent substitution biases vary within the human genome

**DOI:** 10.1186/1471-2105-11-462

**Published:** 2010-09-15

**Authors:** P Andrew Nevarez, Christopher M DeBoever, Benjamin J Freeland, Marissa A Quitt, Eliot C Bush

**Affiliations:** 1 Department of Biology, Harvey Mudd College, Claremont, CA, USA; 2 Department of Biology, Duke University, Durham, NC, USA; 3 Division of Biological Sciences, University of California San Diego, La Jolla, CA, USA; 4 Division of Biology, California Institute of Technology, Pasadena, CA, USA

## Abstract

**Background:**

Models of sequence evolution typically assume that different nucleotide positions evolve independently. This assumption is widely appreciated to be an over-simplification. The best known violations involve biases due to adjacent nucleotides. There have also been suggestions that biases exist at larger scales, however this possibility has not been systematically explored.

**Results:**

To address this we have developed a method which identifies over- and under-represented substitution patterns and assesses their overall impact on the evolution of genome composition. Our method is designed to account for biases at smaller pattern sizes, removing their effects. We used this method to investigate context bias in the human lineage after the divergence from chimpanzee. We examined bias effects in substitution patterns between 2 and 5 bp long and found significant effects at all sizes. This included some individual three and four base pair patterns with relatively large biases. We also found that bias effects vary across the genome, differing between transposons and non-transposons, between different classes of transposons, and also near and far from genes.

**Conclusions:**

We found that nucleotides beyond the immediately adjacent one are responsible for substantial context effects, and that these biases vary across the genome.

## Background

Early models of nucleotide substitution made strong simplifying assumptions, for example assuming that different nucleotides substitute for each other at the same rate [1,2]. Over time it has become clear that many of these assumptions were too strong [3,4]. One assumption that has often been made is that the probability of a substitution at a particular nucleotide position is independent of context, that is the identity of its neighbors. However it is now known that context can substantially bias the substitution process.

The most dramatic example of such substitution bias in vertebrates is the *CG → TG *bias. Typically when a cytosine undergoes deamination it results in a uracil, a situation that is recognized by uracil-DNA glycosylase and repaired by the cell [5]. However, if the cytosine is methylated the result of deamination is thymine. Such cases result in mismatches and lead to an unusually high rate of *C → T *and *G → A *transitions [6]. Because in vertebrates most methylated C residues occur in a CG context, this process causes high rates of *CG → TG *and *CG → CA *transitions, which in turn explains the low frequency of CpG dinucleotides in vertebrate genomes [7-9].

The *CG → TG *bias can be shown by comparative sequence studies using empirical methods [10-12]. Such studies have also revealed several other such biases. These include elevated rates of C*G → A*G and *CG → GG *transversions as well as *TA → CA *transitions, and a tendency toward low substitution rates in purine/pyrimidine tracts [11,12]. Studies in plant chloroplasts found that the proportion of transversions increases with increasing A+T content in flanking sites [13-15]. This effect has also been observed in mitochondrial genomes, and more weakly in nuclear genomes [16], as well as in single nucleotide polymorphisms [17]. It has also been found to extend beyond the adjacent base [14].

In recent years a number of studies have made use of probabilistic models to systematically identify context effects and assess their value in improving model t [4,18-22]. The attraction of these approaches is that they directly model sequence evolution, and provide a framework to access the importance of various context effects. Such studies have identified a large number of bias effects [4,21].

Most studies of substitution bias, both empirical and model-based, have focused on the effects of immediately adjacent bases. There are strong suggestions that more distant biases exist [12,14,23], however to our knowledge no studies have systematically looked for these. Our aim in this study is to do such a systematic examination. The probabilistic models developed in recent years have many advantages, but extending such methods to larger amounts of context represents a substantial technical challenge. This is because of the effects of overlapping windows and the large number of parameters that would need to be estimated.

Here we introduce a simpler empirical method to examine context beyond immediately adjacent bases. Our approach is based on the relative abundance method for studying word frequency bias [24-27]. We have adapted this method to *substitution patterns *rather than words, and applied it to detect context dependent substitution biases in the human lineage after the divergence from chimpanzee. We examined biases in substitution patterns up to 5 bp long and found substantial effects. Most interestingly, we found that bias effects differ in different regions of the genome.

## Methods

### A relative abundance method to characterize substitution biases

Let P be an ancestor-to-descendant substitution pattern (an example is the length 3 pattern *ACT → ATT *). In general, we define a pattern *P *of length *L *as an ancestral sequence of bases paired with a descendant sequence: *P *= *b*_1_*b*_2_...*b_L _*→ *b^′^*_1_*b^′^*_2_...*b^′^_L_*, where *b*_1_, *b_L _*∈ [*A*, *T*, *G*, *C*] and all other *b_i _*∈ [*A*, *T*, *G*, *C*, *N*]. We refer to a pattern containing *N*, which represents any nucleotide, as a gapped pattern (e.g. *ANT → ANT *).

In a dataset of ancestor-descendant alignments, we can define the *proportion *of a pattern *P *to be the fraction of ancestral words that convert to the pattern's descendant sequence:

(1) $$pr(P)=\frac{\text{Num}.\;\;\text{observed}\;b_{1}b_{2}\ldots b_{L}\to \;\;{b^{\prime }}_{1}{b^{\prime }}_{2}\ldots {b^{\prime }}_{L}}{\text{Num}.\;\;\text{observed}\;b_{1}b_{2}...b_{L}}$$

Using this definition, we can calculate the relative abundance *ρ*(*P *) recursively:

(2) $$\rho (P)=\{\begin{array}{cc} pr(P) & \text{if}\;L=1\\ \frac{pr(P)}{\psi (P)} & \text{if}\;L>1 \end{array}$$

where we define *ψ *(*P *) as follows. Let *S_P _*be the set of all gapped and ungapped subpatterns *s *of *P*. Then $\psi (P)=\prod_{s\in S_{P}}\rho (s)$.

In the example from above:

(3) $$\begin{array}{l} \psi (ACT\to ATT)=\rho (A\to A)*\rho (C\to T)\\ \;*\rho (T\to T)*\rho (AC\to AT)\\ \;*\rho (ANT\to ANT)*\rho (CT\to TT) \end{array}$$

*ψ*(*P*) represents the expected proportion of *P *based on the proportions of all its smaller constituent subpatterns. The relative abundance *ρ *is the ratio between the observed proportion of a particular pattern, and this expected proportion. If *ρ*(*ACT → ATT*) were greater than 1 this would suggest a context effect at the 3 bp scale making *ACT → ATT *occur more often than expected. A value less than 1 would suggest an effect making that substitution occur less often than expected.

A natural way to implement eq. 2 is through a dynamic programming approach which avoids redundant calculations. We have developed an algorithm which improves on this by reducing the number of terms to look up. For simplicity of notation, define *B_i _*= *b_i _*→ *b^′^_i_*, so that each *B_i _*represents an ancestor-descendant nucleotide pair. Then *P *= *B*_1_*B*_2_...*B_L_*. If we let *G_P _*be the set of all full-length gapped subpatterns *s *of *P*, we define a new function $\gamma (P)=\prod_{s\in G_{P}}\rho (s)$.We can then calculate relative abundance as:

(4) $$\rho (P)=\{\begin{array}{ll} pr\left(P\right) & \text{if}\;L=1\\ \frac{pr(P)}{\psi (P)} & \text{if}\;L=2\\ \frac{pr(P)pr(B_{2}...B_{L-1})}{pr(B_{1}...B_{L-1})pr(B_{2}...B_{L})\gamma (P)} & \text{if}\;L>2 \end{array}$$

Equations 2 and 4 are equivalent for all^: ^substitution patterns (see proof in Additional file 1). We have implemented Eq. 4 in the C programming language. The source code for this is available on our website http://proconsul.bio.hmc.edu/lp/relabSub.tar.gz. Using the above implementation on current hardware, it takes about 3 minutes and 2 GB of RAM to obtain relative abundance values for all patterns 2-5 bp.

### Estimating the overall impact of context

One would like to know to what degree context biases influence nucleotide substitution at different scales. We not only want to know how under- or over-represented a pattern is (which is captured by eq. 4), but also how important it is to the evolution of genome composition. This depends also on the frequency of the pattern. Thus we define the following measure of the impact of bias from pattern P on genome composition:

(5) Context bias=(ρ(P)−1)*f(P)

Here *f*(*P*) is the frequency of occurrence of the pattern *P *in the data, which is the observed count of the pattern divided by the sum of the counts of all possible gapped and ungapped patterns. For each pattern we calculate *ρ *- 1, and weight this according to *f*(*P*). Because the denominator of *ρ *is the expected proportion of *P *based on the proportions of all its smaller constituent subpatterns, *ρ - *1 is equivalent to (observed *pr*(*P*) - expected *pr*(*P*))/expected *pr*(*P*).

From this we can construct a cumulative measure of bias:

(6) $$\text{Total context bias}=\sum_{P\in [\text{a}11\;\;\text{len}\;\;\text{L}\;pat.]}\left|\rho (P)-1|*f(P\right)$$

where we take the absolute value of *ρ *- 1 for each pattern, weight according to *f*(*P*), and sum across patterns of the same size.

Eq. 6 represents a measure of how much observed proportions at a particular scale deviate from what would be expected if there were no context effects at that scale.

### Assessing the significance of individual patterns

An important aspect of our relative abundance method is that we can identify individual patterns with important contributions to total context bias. To assess the statistical significance of each pattern's context bias contribution, we used a bootstrapping approach. Our datasets each consist of a set of alignment blocks. We sampled with replacement from these until our re-sampled data set had the same number of alignment columns as the original data. From this we calculated both *ρ *(eq. 4) and context bias (eq. 5) for each pattern. We repeated this for 1000 such samples. For each pattern the range of the middle 950 *ρ *and context bias values is the 95% confidence interval.

We also corrected for multiple testing by calculating the false discovery rate for our top patterns. To do this we compared the context bias values for our real data with those for a no bias control to calculate a p-value. We then corrected for multiple testing using the method of Benjamini and Hochberg [28].

### Comparing total context bias in different data sets

One factor that may influence total context bias calculations is sample size. In particular, small data sets can result in artificially high values of context bias (Additional file 2). To control for this we did the following when comparing two alignment data sets. For each data set, we randomly sampled alignment blocks with replacement until the sample contained 50 million alignment columns. Then we calculated context bias for this sample. We repeated this sampling procedure 1000 times.

Doing this for both data sets in the comparison gives two samples of 1000 total context bias values. We then compare the means of each sample with a bootstrap t-test, allowing for unequal variances [29].

### Alignment datasets to assess substitutions in the human lineage

We obtained alignment datasets in transposon and non-transposon regions, and also in regions near and far from genes. We identified transposon regions using RepeatMasker to align SINE, LINE, LTR, and DNA transposons in RepBase to the contigs in NCBI build 36.1 of the human genome [30,31]. From these we removed regions annotated as coding sequence using the UCSC table browser and the knownGenes annotation track [32]. We used the same methods to identify non-transposon noncoding regions. We also used the table browser to identify noncoding regions near and far from genes. Near gene regions were those within 5 kb of a transcription start site for a protein coding gene, while far from gene regions were those at least 50 kb away from a protein coding gene.

We used Galaxy [33] to obtain human-chimpanzee-orangutan alignments [34] over the transposon and non-transposon regions, and regions near and far from genes. The assemblies used were human NCBI build 36.1, chimpanzee March 2006 assembly, and the orangutan March 2007 assembly. From these alignments we removed blocks with fewer than 10 contiguous nongap alignment columns, as well as blocks with more gaps than aligned bases. We then inferred human-chimp ancestral sequences by maximum likelihood using HYPHY [35] and a general time-reversible model of substitution. This model did not allow varying rates across sites.

In the case of our main transposon and non-transposon data sets (sets 1 and 4 in Table 1) we sampled the size of our alignments down to approximately 15% of the genome. This was done for practical reasons having to do with RAM usage in ancestor reconstruction and our algorithm. This sampling was done by randomly choosing 50 kb blocks across the genome, and extracting the coordinates of alignments located within these windows. The other datasets (near and far) did not need to be sampled in this way because they were already small enough.

**Table 1:** Data sets and sizes in alignment columns.

1.	transposon	360,248,252
2.	transposon near gene	53,105,001
3.	transposon far-from-gene	347,985,192
4.	non-transposon	340,461,195
5.	non-transposon near gene	75,324,278
6.	non-transpson far-from-gene	373,326,202
7.	LINE transposons	147,409,103
8.	SINE transposons	106,260,754

Finally, to investigate substitution processes in different types of transposable elements, we extracted subsets of our main transposon data set containing either LINE or SINE transposons. The sizes of all alignment datasets are given in Table 1.

Two of the genomes we are using are drafts (chimpanzee and orangutan). To ensure that sequencing errors would not affect our results, we performed simulations to test the effect of sequencing errors of the magnitude found in these drafts [36]. We randomly introduced errors into our human-chimp-orangutan alignments. The error frequencies we used reflected the human genome's finished status (0.0001 errors/nucleotide), a previously reported error rate for the chimpanzee draft (0.0007 errors/nucleotide) [36], and a similar estimate for orangutan (0.001 errors/nucleotide). We then applied our method to these alignments, and found that errors of this magnitude do not significantly affect our context bias calculations.

It is desirable to use our method with alignments with low divergence. Such alignments make it easier to infer the ancestral sequence, and reduce the possibility that multiple positions in a window have changed (*AAA → AGG *is an example of a window where two base positions have changed.) When multiple positions in a window change it is not possible for us to know what order changes occurred in, and so we cannot know what the exact context was for any individual substitution. This means such windows aren't informative about context biases of the single base substitution process. For this reason, looking at closely related species is preferable, because in such a data set, multiple substitution windows are a small proportion of all windows. Here we have applied our method to alignments between human and the inferred sequence of the human-chimpanzee ancestor. These have a divergence of about 1%.

Because our alignments are between closely related species, ancestor reconstruction is reasonably accurate here, even though we are not using a context-dependent model to do it. The one exception to this is the substitution process at CpG sites. Substitution at such sites is extraordinarily fast. This is by far the strongest bias process at work in these sequences. In this situation, non-context dependent methods of reconstruction can yield some mis-inference of the ancestor even in closely related species such as we are using [37]. We account for this issue in our results below by repeating our analyses with CpG sites removed.

In all our alignment data sets we counted ancestor-descendant patterns and ancestral words for window sizes 1-5 and applied eq. 4.

### No-bias control data sets

To help interpret our results we created no-bias control data sets by taking the inferred human-chimpanzee ancestral sequences from our real data and mutating them according to the observed single-base divergence patterns seen in that real data. For example, in our transposon sequence data, an ancestral C base stays a C 99.1% of the time, and it converts to A, G, and T 0.13%, 0.13%, and 0.60% of the time, respectively. Using these and the corresponding probabilities for other ancestral bases, we mutated the ancestor sequences to create a new descendant sequence. Alignments between this new descendant sequence and the ancestral sequences represent a control data set which has the same ancestral counts and single nucleotide divergence patterns as the real data, but lacks context effects.

## Results

### Context bias effects in transposon insertions in the human lineage

We begin by examining the effects of nucleotide substitution bias acting on transposon insertions in the human lineage after the divergence of chimpanzee. An initial question is how well our results compare with previous results on biases due to adjacent bases. To look at this we considered overrepresented 2 bp patterns identified by our method. Table 2 shows the 15 patterns with the largest relative abundance (*ρ*). Many of these have been previously described [4,11,12,19,21,22,38]. We find that by far the most important bias is the *CG → TG *(or *CG → CA*) pattern, which is highly over-represented (*ρ *= 7.58) [4,11,12,19,21,22,38]. The pattern with the second-highest value of *ρ*, *CG → CT *(*CG → AG*) has also been previously found [12], as has the third, *CG → GG *(*CG → CC*) [12,19,21]. These patterns likely reflect elevated rates of substitution due to cytosine methylation in CpG dinucleotides [11]. The 5th [19], 6th [21], 7th [19], 12th [19] and 13th [11,22] patterns have also been reported previously.

**Table 2:** Top single-substitution patterns of length 2 by relative abundance

Pattern	**Rel. abund**.	**95% Conf. Int**.
1. CG→TG (CG→CA)	7.5809	7.5581 - 7.6049
2. CG→CT (CG→AG)	2.2765	2.2495 - 2.3054
3. CG→GG (CG→CC)	1.9887	1.9605 - 2.0182
4. AT→GT (AT→AC)	1.5444	1.5401 - 1.5492
5. TG→CG (CA→CG)	1.3861	1.3816 - 1.3906
6. AT→TT (AT→AA)	1.3335	1.3244 - 1.3433
7. TA→TT (TA→AA)	1.2402	1.2297 - 1.2504
8. TG→GG (CA→CC)	1.2087	1.1999 - 1.2166
9. GT→TT (AC→AA)	1.2006	1.1920 - 1.2093
10. GT→AT (AC→AT)	1.1903	1.1868 - 1.1943
11. TC→TG (GA→CA)	1.1358	1.1279 - 1.1433
12. TT→TG (AA→CA)	1.1141	1.1073 - 1.1212
13. TA→TG (TA→CA)	1.0990	1.0942 - 1.1039
14. CT→GT (AG→AC)	1.0969	1.0904 - 1.1033
15. GT→CT (AC→AG)	1.0895	1.0816 - 1.0978

The relative abundance metric gives an indication of the degree to which substitution rates vary from expectation and provides a good point of comparison with previous work. While it is informative to know how over- or under-represented a given substitution pattern is, we also want to assess its importance for determining overall genome composition. For this we must also consider how common the pattern is. This is especially true in assessing the importance of larger patterns which occur relatively rarely. To address this we developed a context bias metric (eq. 5) which is the quantity *ρ *- 1 times the frequency of the pattern.

We calculated context bias for all gapped and ungapped patterns from 2-5 bp. Table 3 has the top 50 patterns sorted by context bias, and Additional File 3 has a larger group of patterns which corresponds to the 0.001 FDR group. The top pattern is once again *CG → TG *(context bias = 2.926e-3). Its context bias value is a factor of 10 larger then the next pattern, which is *AT → GT*. The other top entries from Table 2 are also present in Table 3, but in slightly different order because of the weighting by frequency. Note that under-represented patterns are indicated by a negative context bias value.

**Table 3:** Top 50 single-substitution patterns 2-5 bp sorted by context bias

Pattern	Context bias	**95% Conf. Int**.	**Rel. abund**.	**95% Conf. Int**.
1. CG→TG (CG→CA)	2.926e-03	2.907e-03 - 2.945e-03	7.5809	7.5581 - 7.6049
2. AT→GT (AT→AC)	2.148e-04	2.125e-04 - 2.173e-04	1.5444	1.5401 - 1.5492
3. TG→CG (CA→CG)	1.372e-04	1.351e-04 - 1.392e-04	1.3861	1.3816 - 1.3906
4. TG→TA (CA→TA)	-1.025e-04	-1.021e-04 - -1.029e-04	0.6367	0.6341 - 0.6391
5. CT→TT (AG→AA)	-9.985e-05	-9.947e-05 - -1.002e-04	0.6136	0.6110 - 0.6161
6. TNG→CNG (CNA→CNG)	7.681e-05	7.571e-05 - 7.790e-05	1.5309	1.5252 - 1.5365
7. TT→TC (AA→GA)	-7.532e-05	-7.497e-05 - -7.568e-05	0.6385	0.6355 - 0.6413
8. GT→AT (AC→AT)	6.859e-05	6.691e-05 - 7.038e-05	1.1903	1.1868 - 1.1943
9. TC→TT (GA→AA)	-6.753e-05	-6.697e-05 - -6.808e-05	0.7425	0.7399 - 0.7454
10. GG→GA (CC→TC)	-6.618e-05	-6.573e-05 - -6.664e-05	0.7081	0.7049 - 0.7114
11. TT→CT (AA→AG)	-5.828e-05	-5.770e-05 - -5.884e-05	0.7672	0.7641 - 0.7703
12. TC→CC (GA→GG)	-4.745e-05	-4.718e-05 - -4.771e-05	0.6333	0.6296 - 0.6370
13. CG→CT (CG→AG)	3.809e-05	3.676e-05 - 3.950e-05	2.2765	2.2495 - 2.3054
14. CT→CC (AG→GG)	-3.210e-05	-3.144e-05 - -3.275e-05	0.8438	0.8402 - 0.8479
15. GNG→ANG (CNC→CNT)	2.937e-05	2.857e-05 - 3.014e-05	1.1720	1.1682 - 1.1759
16. CG→GG (CG→CC)	2.462e-05	2.358e-05 - 2.564e-05	1.9887	1.9605 - 2.0182
17. AT→TT (AT→AA)	2.382e-05	2.294e-05 - 2.468e-05	1.3335	1.3244 - 1.3433
18. TA→TG (TA→CA)	2.340e-05	2.218e-05 - 2.455e-05	1.0990	1.0942 - 1.1039
19. TNA→TNG (TNA→CNA)	-2.243e-05	-2.214e-05 - -2.270e-05	0.7903	0.7866 - 0.7938
20. GG→AG (CC→CT)	2.236e-05	2.105e-05 - 2.373e-05	1.0655	1.0620 - 1.0692
21. GNG→GNA (CNC→TNC)	1.910e-05	1.839e-05 - 1.976e-05	1.1173	1.1134 - 1.1217
22. TNG→TNA (CNA→TNA)	-1.873e-05	-1.833e-05 - -1.913e-05	0.8679	0.8648 - 0.8713
23. TNT→CNT (ANA→ANG)	-1.844e-05	-1.802e-05 - -1.887e-05	0.8750	0.8715 - 0.8783
24. CNT→CNC (ANG→GNG)	1.733e-05	1.670e-05 - 1.801e-05	1.1524	1.1478 - 1.1575
25. TG→TC (CA→GA)	-1.692e-05	-1.660e-05 - -1.721e-05	0.7668	0.7612 - 0.7723
26. GNT→ANT (ANC→ANT)	-1.673e-05	-1.630e-05 - -1.714e-05	0.8848	0.8814 - 0.8884
27. GT→TT (AC→AA)	1.630e-05	1.552e-05 - 1.709e-05	1.2006	1.1920 - 1.2093
28. TG→GG (CA→CC)	1.610e-05	1.530e-05 - 1.682e-05	1.2087	1.1999 - 1.2166
29. CT→AT (AG→AT)	-1.535e-05	-1.500e-05 - -1.568e-05	0.7949	0.7894 - 0.8006
30. TGG→CGG (CCA→CCG)	-1.482e-05	-1.468e-05 - -1.496e-05	0.7151	0.7116 - 0.7185
31. TA→TT (TA→AA)	1.343e-05	1.273e-05 - 1.411e-05	1.2402	1.2297 - 1.2504
32. TC→TA (GA→TA)	-1.182e-05	-1.149e-05 - -1.214e-05	0.8167	0.8109 - 0.8236
33. GNT→TNT (ANC→ANA)	1.175e-05	1.135e-05 - 1.223e-05	1.2551	1.2474 - 1.2636
34. TTG→CTG (CAA→CAG)	1.173e-05	1.136e-05 - 1.208e-05	1.2408	1.2347 - 1.2469
35. TT→AT (AA→AT)	-1.172e-05	-1.147e-05 - -1.200e-05	0.7803	0.7727 - 0.7868
36. TC→TG (GA→CA)	1.163e-05	1.093e-05 - 1.239e-05	1.1358	1.1279 - 1.1433
37. TNC→TNT (GNA→ANA)	-1.152e-05	-1.099e-05 - -1.204e-05	0.9305	0.9273 - 0.9337
38. GT→GC (AC→GC)	1.090e-05	9.893e-06 - 1.202e-05	1.0588	1.0539 - 1.0639
39. TT→TG (AA→CA)	1.033e-05	9.639e-06 - 1.105e-05	1.1141	1.1073 - 1.1212
40. GGC→GGT (GCC→ACC)	9.916e-06	9.571e-06 - 1.026e-05	1.2075	1.2018 - 1.2140
41. GC→GG (GC→CC)	-9.655e-06	-9.383e-06 - -9.932e-06	0.7853	0.7772 - 0.7938
42. CT→GT (AG→AC)	9.557e-06	8.870e-06 - 1.027e-05	1.0969	1.0904 - 1.1033
43. TT→TA (AA→TA)	-9.479e-06	-9.164e-06 - -9.822e-06	0.8338	0.8268 - 0.8413
44. TC→GC (GA→GC)	-9.314e-06	-9.093e-06 - -9.545e-06	0.7587	0.7509 - 0.7677
45. GNG→CNG (CNC→CNG)	9.304e-06	8.933e-06 - 9.707e-06	1.2409	1.2320 - 1.2497
46. GNNG→GNNA (CNNC→TNNC)	9.232e-06	8.864e-06 - 9.596e-06	1.1145	1.1104 - 1.1185
47. GG→TG (CC→CA)	-8.903e-06	-8.530e-06 - -9.274e-06	0.8544	0.8467 - 0.8619
48. TNNG→TNNA (CNNA→TNNA)	-8.708e-06	-8.482e-06 - -8.924e-06	0.8822	0.8787 - 0.8856
49. TG→TT (CA→AA)	-8.545e-06	-8.105e-06 - -9.015e-06	0.9046	0.8987 - 0.9102
50. GG→CG (CC→CG)	-7.914e-06	-7.543e-06 - -8.279e-06	0.8664	0.8596 - 0.8745

An interesting point is that several longer patterns show up in our results. The 3 bp pattern with the largest context bias is entry 6, *TNG → CNG *(context bias = 7.681e-5, *ρ *= 1.53). This is within a factor of 40 of the absolute value of context bias for *CG → TG*, and within a factor of 3 of that for *AT → GT *, the largest non-CG 2 bp pattern. There are also 4 bp patterns, and the largest of these, *GNNG → GNNA *is approximately a factor of 300 smaller than *CG → TG *and a factor of 25 smaller than *AT → GT*. In Additional File 3, there are even some 5 bp patterns. The largest of these is *TNNNT → TNNNC *(context bias = -3.49e-6, *ρ *= 0.90). The longer patterns that our context bias metric identifies are largely gapped patterns. This is because such patterns occur more frequently, and our context bias measure is designed to take frequency into account. However there are a few ungapped 3 bp patterns which appear in the top group, for example, *TGG → CGG *and *TTG → CTG*, entries 30 and 34 in Table 3. In Additional File 3 there is even an ungapped 4 bp pattern, *CGCC → TGCC *(context bias = -2.80e-06, *ρ *= 0.81) which is entry 133.

**Figure 1:**
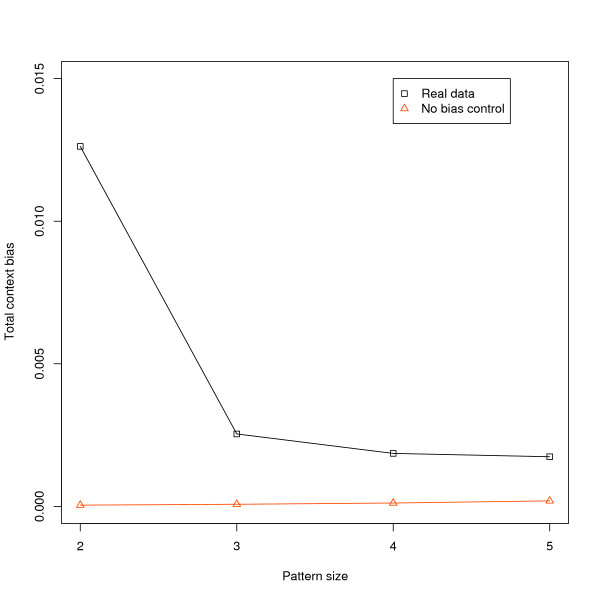
**Total context bias in transposon sequences along the human lineage after the divergence from chimpanzee**. Real data is shown in black, while values for a corresponding no bias control are in orange. Context bias is greatest at the 2 bp scale, with *CG → TG *having the largest contribution. Total bias then drops, but remains level in 3-5 bp patterns rather than continuing to decrease.

One of the novel aspects of our method is it offers a way to measure the aggregate effects of bias at a particular size scale. We do this by summing the absolute value of our context bias measure across all patterns at a particular size (eq. 6). Figure 1 and Table 4 present our results for human lineage data at 2-5 bp as well as no-bias controls. Bias effects in the real data are substantially higher than in the no-bias controls. It can also be seen that the overall impact of context bias drops off substantially from 2-3 bp. The 2 bp total context bias value is about a factor of 5 larger than the value for 3. However, from 3 to 5 bp the value stays relatively steady.

**Table 4:** Total context bias in transposons and non-repeats, and subsets near or far from genes.

	2 bp	3 bp	4 bp	5 bp
**Real data**				
Transposon	1.2622e-2	2.5405e-3	1.8575e-3	1.7409e-3
Transposon, near	1.2981e-2	2.7622e-3	2.2495e-3	2.4520e-3
Transposon, far	1.1799e-2	2.4298e-3	1.6990e-3	1.3874e-3
Non-repeat	9.0816e-3	2.1532e-3	1.3989e-3	1.0385e-3
Non-repeat, near	7.5286e-3	2.1473e-3	1.4786e-3	1.2069e-3
Non-repeat, far	9.3789e-3	2.2102e-3	1.4290e-3	1.0287e-3
LINEs	9.7298e-3	2.1798e-3	1.4683e-3	1.1465e-3
SINEs	1.8042e-2	3.6693e-3	3.6117e-3	4.3391e-3
				
**No-bias controls**				
Transposon	4.3907e-5	7.4125e-5	1.1806e-4	1.9323e-4
Transposon, near	7.4565e-5	1.7935e-4	3.1243e-4	5.0755e-4
Transposon, far	3.7193e-5	7.4758e-5	1.2081e-4	2.0596e-4
Non-repeat	3.1367e-5	7.1670e-5	1.1779e-4	1.9054e-4
Non-repeat, near	8.3869e-5	1.3339e-4	2.6857e-4	4.1832e-4
Non-repeat, far	4.1651e-5	7.3881e-5	1.2627e-4	1.8502e-4
LINEs	5.6367e-5	1.0365e-4	1.8279e-4	2.8490e-4
SINEs	7.2458e-5	1.4746e-4	2.4695e-4	3.9894e-4

A concern with our method is that context bias values could be affected by sample size. To address this, we re-sampled the full human transposon data at a range of sample sizes, and calculated context bias for each sample. We then repeated this for the corresponding no bias control data (Additional File 2). At very small sample sizes, we find that median context bias values in both real and no bias data are elevated due to stochastic effects. However, at the sample sizes used in this analysis, stochastic effects are negligible.

### Context bias in transposon vs. non-transposon sequences

One question we can ask with this method is whether bias effects vary across the genome. We start by comparing transposon vs. non-transposon sequences. At the 2 bp scale differences in substitution bias have been observed between these two sequence types. These are due to the differences in methylation level between transposons and non-transposons [39]. Our method allows us to look for similar effects at larger scales.

**Figure 2:**
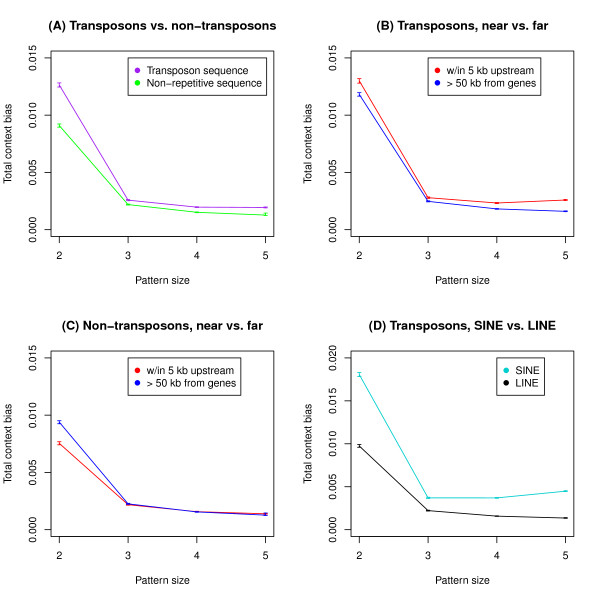
**Comparison of context bias in different types of sequence**. Bars represent 95% confidence intervals. At pattern sizes from 2-5 bp, context bias is greater in transposon sequence than in non-repetitive sequence (A). Among transposons, context bias is greater near genes than far from them (B). Conversely, context bias is never significantly greater near genes in non-repetitive sequence, with markedly higher context bias far from genes for 2 bp patterns (C). Context bias also differs between the most common classes of transposons, LINEs and SINEs (D).

To do this we generated a set of non-transposon sequences, covering approximately 15% of the human genome, and used human-chimpanzee-orangutan alignments to infer the human-chimpanzee ancestral sequence. We then applied eq. 6 to both transposon and non-transposon sets of alignments. As illustrated in Figure 2A and Table 4, we find higher total context bias in transposon sequence than in non-transposon sequence. This difference extends over the whole size range from 2-5 bp, and is statistically significant (bootstrap-t test, *p *< 0.001).

The *CG → TG *effect is likely to contribute a significant part of the transposon vs. non-transposon difference at 2 bp. In fact, it could potentially contribute to effects at larger pattern sizes, if the magnitude of the *CG → TG *effect were correlated with some attribute like the G+C content of nearby bases [40]. To control for this we calculated context bias for our alignment data, removing patterns where the ancestor contains a CpG dinucleotide. This eliminates the possibility that context effects at the 3-5 bp scale might be acting via the rate of cytosine deamination at CpG sites. After removing all patterns with an ancestral CpG (Additional File 4A) we find that transposon sequences still have a significantly larger degree of context bias than non-transposon sequences.

Another concern is that transposon vs. non-transposon difference in our total context bias measure might be due to differences in pattern composition, rather than differences in bias. Comparison with our no-bias controls partially alleviates this concern. To be even more confident, we repeated our analysis, calculating an unweighted version of eq. 6 over all single base substitution patterns P. In the unweighted version we replace the *f*(*P*) term with with 1/N, where N is the total number of patterns. Using this measure we get similar results. Bias is larger in transposons than non-transposon regions for all pattern sizes. (Additional File 5).

In order to better understand the source of this context bias difference, we compared individual patterns from transposon and non-repetitive alignments. The difference between transposon and non-repetitive datasets is due to a relatively small fraction of patterns which have different values in the two (Additional File 6).

### Context bias varies near and far from genes

A second way in which genomic location may affect substitution bias is based on proximity to genes. Evidence from the rate of change of word frequencies in DNA suggests such effects exist [41].

To examine this we obtained alignment data from regions near and far from genes. The near-gene data sets consist of alignments from within 5 kb upstream of transcription start sites of protein coding genes. The far-from-gene data consist of alignments located more than 50 kb away from protein coding genes. We did this for both our transposon and non-transposon sequence. We find that the amount of substitution bias varies significantly based on proximity to genes. Moreover, the nature of this effect is different in transposons and non-repetitive sequence.

In transposon sequences, total context bias is significantly greater near genes than far from them at all pattern sizes (Figure 2B). In non-repetitive sequences, context bias at the 2 bp level is greater far from genes than near them, the opposite of what is observed in transposons (bootstrap-t test, *p *< 0.001). At 3-5 bp, near and far are roughly comparable (Figure 2C). When we remove all CpG patterns as above, these results remain the same (Additional File 4B and 4C).

An examination of the source of these total context bias differences shows that they are due to a comparatively small number of patterns (Additional File 6).

### Comparison of LINE and SINE transposons

Another question is whether substitution bias varies between transposon types. Such variation could explain our near-gene far-from-gene observations in transposon sequence, because the distribution of transposons is known to vary based on proximity to genes. To look at this we extracted LINE and SINE derived sequences from our full transposon dataset, and compared context bias between these two transposon classes.

SINE elements genome-wide have substantially higher context bias than LINEs at all pattern sizes (Figure 2D), a difference which remains when CpG patterns are removed (Additional File 4D). As has been previously reported, the distribution of Alu SINEs is correlated with gene density, which is not the case for LINEs [42]. The localization of these common SINEs near genes, along with their elevated context bias compared to LINEs, may help explain the difference in bias at different distances from genes.

## Discussion

Here we have presented a method to examine context-dependent nucleotide substitution biases. Our method is based on the relative abundance method for word frequencies and allows us to disentangle context effects at different size scales. With it we can systematically examine context biases from beyond the adjacent base, something which had not been possible previously. We applied our method to the human lineage after the divergence from chimpanzee, measuring context effects in substitution patterns from 2 to 5 bp, and finding significant effects at all sizes.

Our results for 2 bp patterns are broadly consistent with previous studies of context-dependent substitution [4,11,12,19,21,22,38]. For example, Arndt and Hwa [19] used a probabilistic model to look at context effects due to the immediately adjacent base. They studied substitution patterns in AluSx SINE insertions in the human lineage, which represents a significantly smaller dataset than ours. Despite differences in dataset and methodology, all three of the patterns they identify as over-represented are among our top patterns. These are *CG → TG*, *CG → GG*, and *TT → TG*, and are our first, third and twelfth entries respectively (Table 2).

We identified a number of 3 and 4 bp patterns with large bias effects which may have a significant impact on genome composition. The largest number of these are gapped patterns such as *TNG → CNG*. Gapped patterns occur at higher frequency than ungapped, and for this reason are more likely to impact genome composition. However there are also some ungapped 3 bp patterns which appear in Table 3. Interestingly all three ungapped patterns have substitutions on the end. This means that they would not have been found by previous methods which looked at the effect of both adjacent bases on substitutions at a central site (e.g. [21]).

The fact that some gapped 3 bp patterns appear at the top of Table 3 suggests that such patterns may be worth incorporating into probabilistic substitution models which consider context. In some circumstances (i.e. when the dataset is large) adding parameters for these processes may significantly improve models.

In addition to identifying over- and under-represented substitution patterns, our method also allows us to estimate the aggregate effects of context bias at a particular size scale. We found that these drop substantially from 2 to 3 bp, but then level off from 3-5 bp where they remain significantly larger than the no bias controls. The drop off from 2 to 3 is not unexpected. In part it reflects the strong influence of the CpG effect. However the CpG effect does not account for all of the difference between 2 and 3 bp, as can be seen when we remove the CpG effects (Additional File 4). This shows that nearest neighbor bias effects in general are very strong compared to more distant effects. What is a little unexpected is the fact that total context bias at 5 bp is similar to that at 3. When we imagine the kind of molecular processes which could produce these biases, it seems reasonable that influence would drop off with distance. But at least between 3 and 5 bp this appears not to be the case.

The most interesting aspect of our results is the finding that different types of sequence are subject to different context dependent biases. In particular, sequences derived from transposon insertions are subject to different and greater biases than non-repetitive sequences. This likely reveals something about the underlying genomic processes affecting substitution in these regions.

One way to explain regional variation in context dependent substitution processes is via selective mechanisms. Purifying selection disfavors some mutations from becoming fixed, which can produce substitution biases. Biases created in this way might vary across the genome since the density of functional sequences varies across the genome. However, our data aren't entirely consistent with the selective explanation. Ancestral transposon insertions are a fairly neutral category of sequence. If selection were the main explanation for bias, we wouldn't expect transposon derived sequences to have more bias than non-repetitive sequences. But that is what our data show (Figure 2A).

Mutational explanations offer an alternative. At the 2 bp scale, there is a mutational explanation which is very consistent with our data and previous work. This explanation is that that differences in context bias result from variation in the methylation of CpG dinucleotides. Consider our results at 2 bp. First, we observed that context bias is greater in transposons than non-repetitive sequences (Figure 2A). A large part of the difference is due to the fact that CpGs are more likely to be methylated in transposons [39,43]. Indeed, when we remove alignment columns with ancestral CpGs, the difference in total context bias between transposon and non-repetitive sequence at 2 bp is reduced substantially. Second, for non-transposon sequences the bias at 2 bp is significantly less near genes than far from them (Figure 2E). This can be explained by the fact that CpG islands are more common near genes [44]. In such regions, most Cs are unmethylated, and are thus much less likely to undergo a transition to T. This will tend to reduce our context bias measure.

In transposon sequences the near gene vs. far-from-gene result is reversed; total context bias is greater near genes. This result may be due to non-uniform distribution of transposable elements across the genome. Alu elements, the most common SINEs, are associated with gene density [42], while LINEs are not. We have also found that context bias in SINEs is higher than in LINEs (Figure 2D). These considerations suggest that the near gene vs. far-from-gene differences in context bias may be explained by the transposon distribution at different distances from genes.

We also observed substantial context effects at scales larger than 2 bp. These share some important similarities with the results at 2 bp. Most important is the transposon vs. non-transposon difference. Total context bias in transposon sequence is greater at 3-5 bp (Figure 2A). Also, just as at 2 bp, the effect of gene proximity is different in the two types of sequence. Transposon sequences have greater total context bias near genes than far from them, which may be due to differences transposon distribution near and far from genes. Non-repetitive sequences lack this trend.

The bias effects we observe at 3-5 bp may represent novel genomic processes. One possibility is the existence of defense mechanisms against genomic parasites, which as a byproduct of their activity produce substitution biases. Such mechanisms would need to operate differently on transposon vs. non-transposon sequence, and also on different transposon classes. The existence of such mechanisms could explain some of the similarities we see to the CpG methylation dependent bias; one of the important functions of CpG methylation is defense against transposons [43,45].

Our method has allowed us to systematically examine substitution biases beyond the adjacent base, and shown that such biases do exist. However it has several limitations. First, it is sensitive to stochastic variation. As we move to larger pattern sizes, and individual patterns become rarer, stochastic variation in our estimates of context bias increases. We found that this problem became important at 6 bp and particularly at 7 bp. For this reason in this study we focused on pattern sizes 2-5 bp, where stochastic effects are not a serious problem (Additional File 2). Our approach also has the limitations associated with an empirical rather than model based approach. Bias processes which are extremely fast, such as the CpG process, are not handled well by such approaches. In addition, the need to reconstruct the ancestral sequence accurately limits us to using alignments from closely related species.

There are several avenues to pursue in the future. One is to develop a model based approach to this problem, which would alleviate some of the limitations mentioned above. The present study provides a foundation for that by giving an indication of the kinds of processes which are likely to be important. Another future direction is to look for better ways to compare bias in different sequence regions. Here we have used total context bias. It might be possible to do this in a more fine-grained way, by developing distance measures which take into account differences in context bias (or *ρ*) values for individual patterns. Such comparisons could be carried out between a much wider range of sequence types, including introns, untranslated regions, and coding sequence. Such methods would also make it possible to compare substitution bias in different lineages, such as in the chimpanzee vs. the human lineage.

## Conclusions

We developed a method to systematically characterize context dependent nucleotide substitution bias, and applied it to 2-5 bp patterns. We find significant effects at all sizes, with the largest effects at 2 bp. Our most interesting result is that context effects vary across the human genome. In particular there are significant differences between transposon-derived and non-transposon sequence. Transposon sequences have more bias at all scales from 2-5 bp. In addition, bias effects differ between transposon classes as well as near and far from genes. The variation at the 2 bp scale can likely be explained by variation in CpG methylation. But at larger scales it may be due to novel processes, possibly processes related to genomic defense against transposons.

## Authors' contributions

PAN carried out the analysis and wrote the manuscript. CMD, BAF and MAQ carried out the analysis. ECB designed the project, carried out the analysis, and wrote the manuscript. All authors read and approved the final paper.

## Supplementary Material

Additional file 1 **Proof of relative abundance algorithm by mathematical induction**. PDF file displaying Proof of relative abundance algorithm by mathematical induction. Click here for file

Additional file 2 **Effect of sample size on total context bias calculation**. To determine the effect of stochastic variation in pattern frequencies on our context bias estimates, we calculated total context bias at a variety of sample sizes. We repeatedly sampled with replacement from from our full transposon data set. We took a total of 5380 samples at 120 sample sizes. Here we have plotted the median total context bias at each sample size against sample size. For comparison we've also included the no-bias controls. At low sample sizes stochastic effects elevate context bias. This effect diminishes rapidly with increasing amounts of data. Click here for file

Additional file 3 **Table of top context bias values for 2-5 bp single substitution patterns**. We calculated context bias values for all single substitution 2-5 bp patterns for our transposon dataset, and for 10 corresponding no bias control data sets. We used the no-bias controls to determine a p-value for each pattern in the real data. (The no-bias controls tell us how likely are we to get a score this high or higher if there were in reality no bias.) We then used the method of Benjamini and Hochberg, 1995 to identify the set of patterns with a false discovery rate of 0.001. Those patterns are given in this table. Click here for file

Additional file 4 **Comparison of context bias after removing CpG-containing patterns**. One possible explanation for observed differences in context bias is that the methylation process that produces biases at 2 bp is also influenced by context at larger scales. To address this, we calculated context bias for each data set in Figure 2 while excluding substitution patterns including an ancestral CpG. We find that the effects at 3-5 bp remain, which suggests that bias at these scales is not working via the rate of cytosine deamination at CpG sites. Click here for file

Additional file 5 **Unweighted total context bias in tranposons and non-repetitive sequence**. Differences in total context bias between transposons and non-transposons might be due to variation in pattern frequencies rather than difference in the substitution process. To address this, we calculated an unweighted version of eq. 5 across all single-substitution patterns at each pattern size. To do this we simply replaced the term f(P) in eq. 5 with the term 1/N, where N is the total number of patterns. With this new measure, as with total context bias, we find that transposons have more bias than non-transposon sequence at all sizes. Click here for file

Additional file 6 **Distribution of context bias differences between human lineage data sets**. We found that total context bias differs between different types of sequence, for example between transposons and non-repetitive sequences. One question we would like to answer is what is the origin of this difference. It turns out it is not due to patterns which are unique in one data set or the other. Another question is whether the differences is due to differences in a few shared patterns, or many. Here we compare context bias values for patterns which are shared. For example, in (A) we are looking at 2 bp patterns. We calculate the value of transposon minus non-repetitive for each of these. We then sort large to small, and plot them according to their rank. The y value of this plot is the cumulative value of context bias difference. The horizontal line represents the total context bias value for all patterns. As can be seen, most of the final total context bias value is due to a few patterns which differ greatly in transposons and non-repetitive sequence. A-D represent transposon vs. non-repetitive for 2-5 bp, E-H represent near-far for transposon sequences, and I-L represent far-near for non-repetitive sequences. Click here for file
