# Supplementary material for: Context dependent substitution biases vary within the human genome
Source: BMC Bioinformatics. 2010 Sep 15;11:462. doi: 10.1186/1471-2105-11-462 (PMC2945941; doi:10.1186/1471-2105-11-462)
Supplement: Additional file 1 — Proof of relative abundance algorithm by mathematical induction. PDF file displaying Proof of relative abundance algorithm by mathematical induction. [file 1471-2105-11-462-S1.PDF]

Calculating relative abundance values

Let P be a ancestor-to-descendant substitution pattern with length L :

$P = b_1 b_2 \ldots b_L \to b_1' b_2' \ldots b_L'$

Where $b_1, b_L \in [A, T, G, C]$ and all other $b_i \in [A, T, G, C, N]$. We can write each ancestor-descendant nucleotide pair as $B_i = b_i \to b_i'$. Then

$P = B_1 B_2 \ldots B_L$

Given a set of ancestor-descendant alignments, the proportion of P is the fraction of ancestral words that convert to the appropriate descendant sequence:

$pr(P) = \frac{\text{Number of observed } b_1 b_2 ... b_L \to b_1' b_2' ... b_L'}{\text{Number of observed } b_1 b_2 ... b_L}$

The normal recursive method for calculating relative abundance is:

$$\rho(P) = \begin{cases} pr(P) & \text{if } L = 1 \\ \frac{pr(P)}{\psi(P)} & \text{if } L > 1 \end{cases} \tag{1}$$

where $\psi(P)$ is the product of all elements in S_P, the set of all subpatterns s of P:

$$\psi(P) = \prod_{s \in S_P} \rho(s)$$

S_P contains all gapped and ungapped subpatterns, with N representing any base.

We have proposed a different method of calculating relative abundance, which we refer to as the "seg algorithm." If we let G_P be the set of all full-length gapped subpatterns s of P, define a new function γ:

$$\gamma(P) = \prod_{s \in G_P} \rho(s)$$

The seg algorithm is:

$$\rho(P) = \begin{cases} pr(P) & \text{if } L = 1 \\ \frac{pr(P)}{\psi(P)} & \text{if } L = 2 \\ \frac{pr(P) pr(B_2 ... B_{L-1})}{pr(B_1 ... B_{L-1}) pr(B_2 ... B_L) \gamma(P)} & \text{if } L > 2 \end{cases} \tag{2}$$

The algorithms are the same for patterns of length 1 or 2 by definition. We can demonstrate by mathematical induction that they are also equal for all patterns with $L > 2$.

Justification of the "seg algorithm"

Proof. Suppose that P is a substitution pattern with $L = 3$. Then from Equation 1, we have

$$\begin{aligned}
\rho(P) &= \frac{pr(B_1B_2B_3)}{\psi(B_1B_2B_3)} \\
&= \frac{pr(B_1B_2B_3)}{pr(B_1)pr(B_2)pr(B_3)\rho(B_1B_2)\rho(B_1NB_3)\rho(B_2B_3)} \\
&= \frac{pr(B_1B_2B_3)}{pr(B_1)pr(B_2)pr(B_3)[\frac{pr(B_1B_2)}{pr(B_1)pr(B_2)}][\frac{pr(B_1NB_3)}{pr(B_1)pr(B_3)}][\frac{pr(B_2B_3)}{pr(B_2)pr(B_3)}]} \\
&= \frac{pr(B_1B_2B_3)pr(B_1)pr(B_2)pr(B_3)}{pr(B_1B_2)pr(B_2B_3)pr(B_1NB_3)}
\end{aligned}$$

Similarly, using the same pattern P and Equation 2,

$$\begin{aligned}
\rho(P) &= \frac{pr(B_1B_2B_3)pr(B_2)}{pr(B_1B_2)pr(B_2B_3)\gamma(B_1B_2B_3)} \\
&= \frac{pr(B_1B_2B_3)pr(B_2)}{pr(B_1B_2)pr(B_2B_3)\rho(B_1NB_3)} \\
&= \frac{pr(B_1B_2B_3)pr(B_2)}{pr(B_1B_2)pr(B_2B_3)[\frac{pr(B_1NB3)}{pr(B_1)pr(B_3)}]} \\
&= \frac{pr(B_1B_2B_3)pr(B_1)pr(B_2)pr(B_3)}{pr(B_1B_2)pr(B_2B_3)pr(B_1NB_3)}
\end{aligned}$$

Thus, Equations 1 and 2 are equal for patterns with $L = 3$.

Inductive step. Suppose that Eq. 1 is equal to Eq. 2 for patterns of length $n > 2$. Then for a pattern $P = B_1 \ldots B_n$, combining the equations gives us the following inductive hypothesis:

$$\rho(P) = \frac{pr(P)}{\psi(P)} = \frac{pr(P)pr(B_2 \ldots B_{n-1})}{pr(B_1 \ldots B_{n-1})pr(B_2 \ldots B_n)\gamma(P)} \tag{3}$$

Assuming it works for P, we want to prove that this holds for a pattern P^+, with length $n + 1$. Starting with the right side of Eq. 3, for P^+ we have:

$$\begin{aligned}
\rho(P^+) &= \frac{pr(P^+)pr(B_2...B_n)}{pr(B_1...B_n)pr(B_2...B_{n+1})\gamma(P^+)} \\
&= \frac{pr(P^+)pr(B_2...B_n)}{pr(P)pr(B_2...B_{n+1})\gamma(P^+)}
\end{aligned} \tag{4}$$

Solving Eq. 3 for $\gamma(P)$ gives

$$\gamma(P) = \frac{\psi(P)pr(B_2 \ldots B_{n-1})}{pr(B_1 \ldots B_{n-1})pr(B_2 \ldots B_n)} \tag{5}$$

Then from Eq. 4, using the expression for $\gamma(P)$ in Eq. 5 leads to

$$\begin{aligned}
\frac{pr(P^+)pr(B_2...B_n)}{pr(P)pr(B_2...B_{n+1})\gamma(P^+)} &= \frac{pr(P^+)pr(B_2...B_n)}{pr(P)pr(B_2...B_{n+1})[\frac{\psi(P^+)pr(B_2...B_n)}{pr(B_1...B_n)pr(B_2...B_{n+1})}]} \\
&= \frac{pr(P^+)pr(B_1...B_n)}{pr(P)\psi(P^+)} \\
&= \frac{pr(P^+)pr(P)}{pr(P)\psi(P^+)} \\
&= \frac{pr(P^+)}{\psi(P^+)}
\end{aligned}$$

We have shown that the two algorithms are equal for patterns of length 1, 2, and 3. We have also shown by induction that if they are equivalent for patterns of length $n > 2$, then they must also be equal for patterns of length $n + 1$. As such, we conclude that the algorithms are equivalent for substitution patterns of all lengths.

□
