# Supplementary figures and images for: Context dependent substitution biases vary within the human genome
Source: BMC Bioinformatics. 2010 Sep 15;11:462. doi: 10.1186/1471-2105-11-462 (PMC2945941; doi:10.1186/1471-2105-11-462)

**2 bp**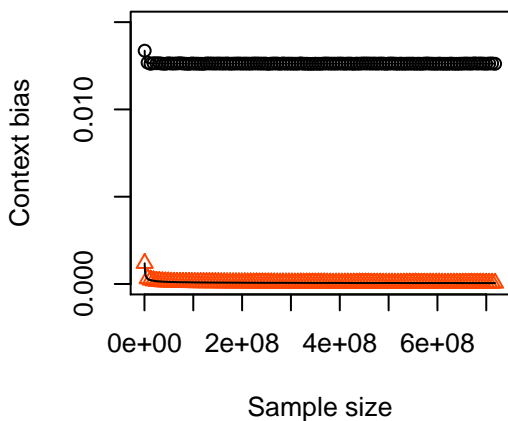**3 bp**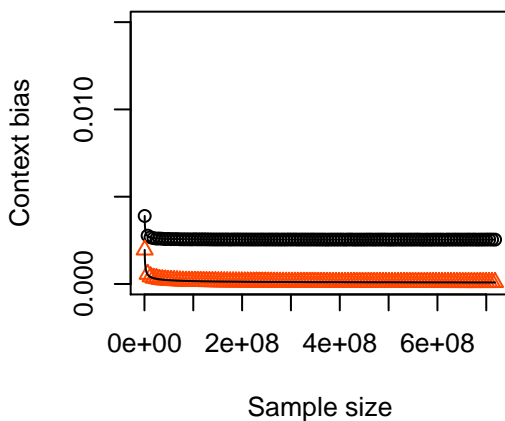**4 bp**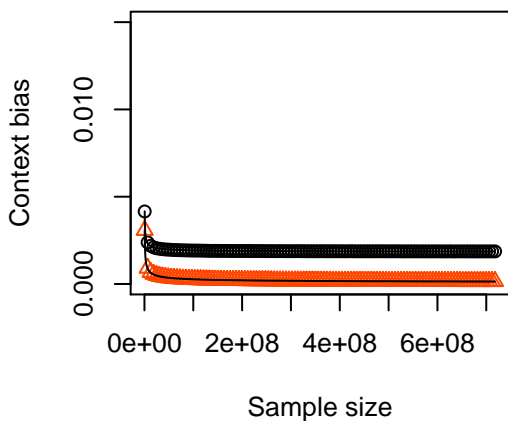**5 bp**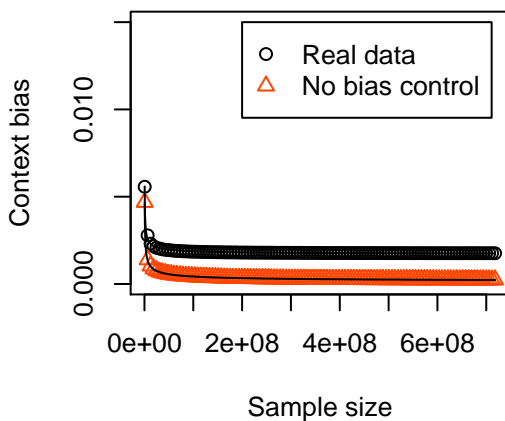

Supplement: Additional file 2 — Effect of sample size on total context bias calculation. To determine the effect of stochastic variation in pattern frequencies on our context bias estimates, we calculated total context bias at a variety of sample sizes. We repeatedly sampled with replacement from from our full transposon data set. We took a total of 5380 samples at 120 sample sizes. Here we have plotted the median total context bias at each sample size against sample size. For comparison we've also included the no-bias controls. At low sample sizes stochastic effects elevate context bias. This effect diminishes rapidly with increasing amounts of data. [file 1471-2105-11-462-S2.PDF]
