# Supplementary material for: Context dependent substitution biases vary within the human genome
Source: BMC Bioinformatics. 2010 Sep 15;11:462. doi: 10.1186/1471-2105-11-462 (PMC2945941; doi:10.1186/1471-2105-11-462)
Supplement: Additional file 4 — Comparison of context bias after removing CpG-containing patterns. One possible explanation for observed differences in context bias is that the methylation process that produces biases at 2 bp is also influenced by context at larger scales. To address this, we calculated context bias for each data set in Figure 2 while excluding substitution patterns including an ancestral CpG. We find that the effects at 3-5 bp remain, which suggests that bias at these scales is not working via the rate of cytosine deamination at CpG sites. [file 1471-2105-11-462-S4.PDF]

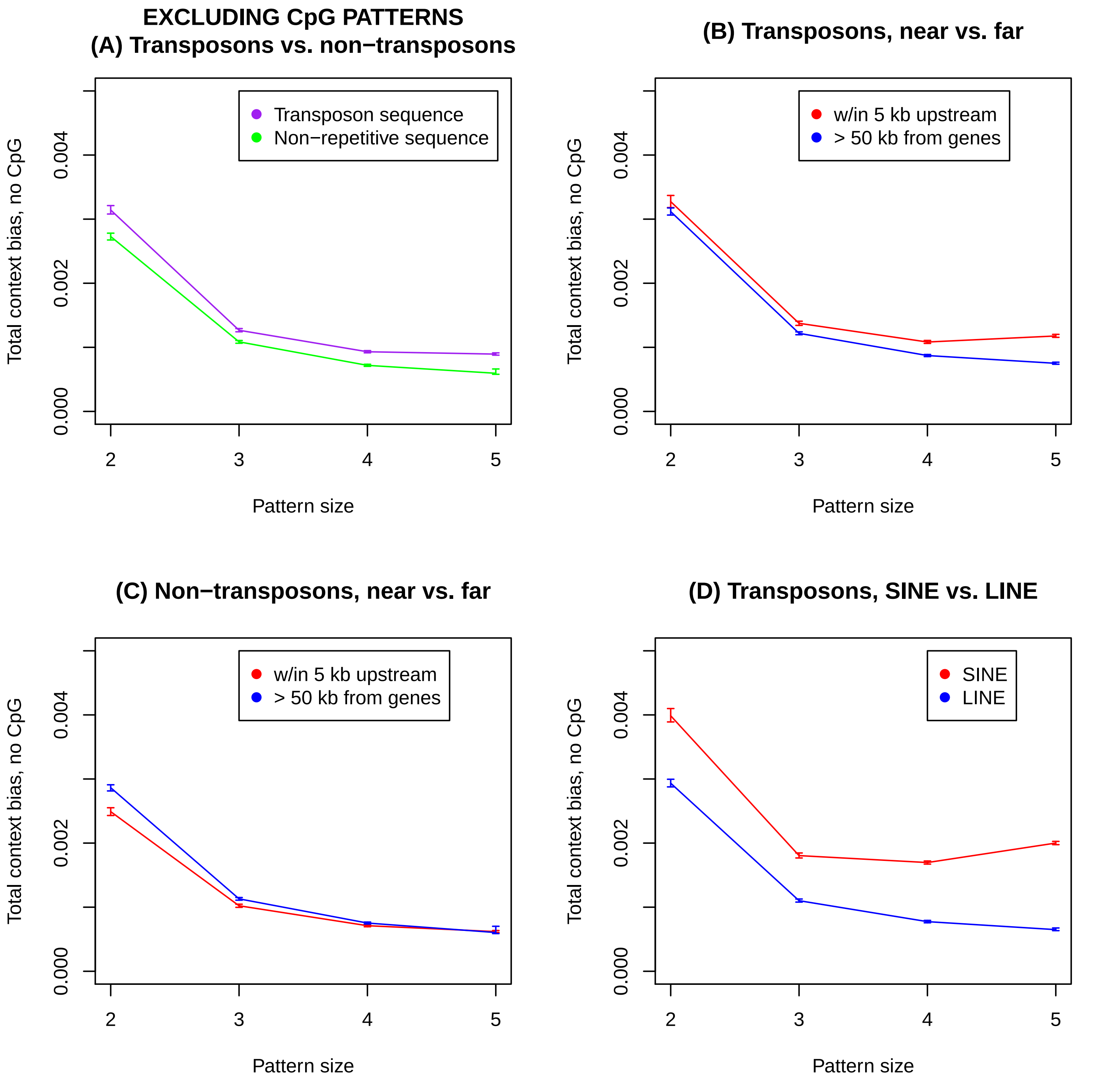
EXCLUDING CpG PATTERNS
(A) Transposons vs. non−transposons
Transposon sequence
Non−repetitive sequence
Total context bias, no CpG
0.000
0.002
0.004
2
3
4
5
Pattern size
(B) Transposons, near vs. far
w/in 5 kb upstream
> 50 kb from genes
Total context bias, no CpG
Pattern size
(C) Non−transposons, near vs. far
w/in 5 kb upstream
> 50 kb from genes
Total context bias, no CpG
Pattern size
(D) Transposons, SINE vs. LINE
SINE
LINE
Total context bias, no CpG
Pattern size
