# Supplementary material for: Context dependent substitution biases vary within the human genome
Source: BMC Bioinformatics. 2010 Sep 15;11:462. doi: 10.1186/1471-2105-11-462 (PMC2945941; doi:10.1186/1471-2105-11-462)
Supplement: Additional file 5 — Unweighted total context bias in tranposons and non-repetitive sequence. Differences in total context bias between transposons and non-transposons might be due to variation in pattern frequencies rather than difference in the substitution process. To address this, we calculated an unweighted version of eq. 5 across all single-substitution patterns at each pattern size. To do this we simply replaced the term f(P) in eq. 5 with the term 1/N, where N is the total number of patterns. With this new measure, as with total context bias, we find that transposons have more bias than non-transposon sequence at all sizes. [file 1471-2105-11-462-S5.PDF]

Additional file 5 - Average difference from expected ρ

	2 bp	3 bp	4 bp	5 bp
**Real data**				
Transposon	3.5082e-1	1.0289e-1	7.1793e-2	6.5955e-2
Non-repeat	3.3493e-1	1.0073e-1	6.2685e-2	4.6306e-2
**No-bias controls**				
Transposon	2.9042e-3	5.4077e-3	9.1668e-3	1.6507e-2
Non-repeat	2.1932e-3	5.7114e-3	9.6713e-3	1.7038e-2
