# Supplementary material for: Context dependent substitution biases vary within the human genome
Source: BMC Bioinformatics. 2010 Sep 15;11:462. doi: 10.1186/1471-2105-11-462 (PMC2945941; doi:10.1186/1471-2105-11-462)
Supplement: Additional file 6 — Distribution of context bias differences between human lineage data sets. We found that total context bias differs between different types of sequence, for example between transposons and non-repetitive sequences. One question we would like to answer is what is the origin of this difference. It turns out it is not due to patterns which are unique in one data set or the other. Another question is whether the differences is due to differences in a few shared patterns, or many. Here we compare context bias values for patterns which are shared. For example, in (A) we are looking at 2 bp patterns. We calculate the value of transposon minus non-repetitive for each of these. We then sort large to small, and plot them according to their rank. The y value of this plot is the cumulative value of context bias difference. The horizontal line represents the total context bias value for all patterns. As can be seen, most of the final total context bias value is due to a few patterns which differ greatly in transposons and non-repetitive sequence. A-D represent transposon vs. non-repetitive for 2-5 bp, E-H represent near-far for transposon sequences, and I-L represent far-near for non-repetitive sequences. [file 1471-2105-11-462-S6.PDF]

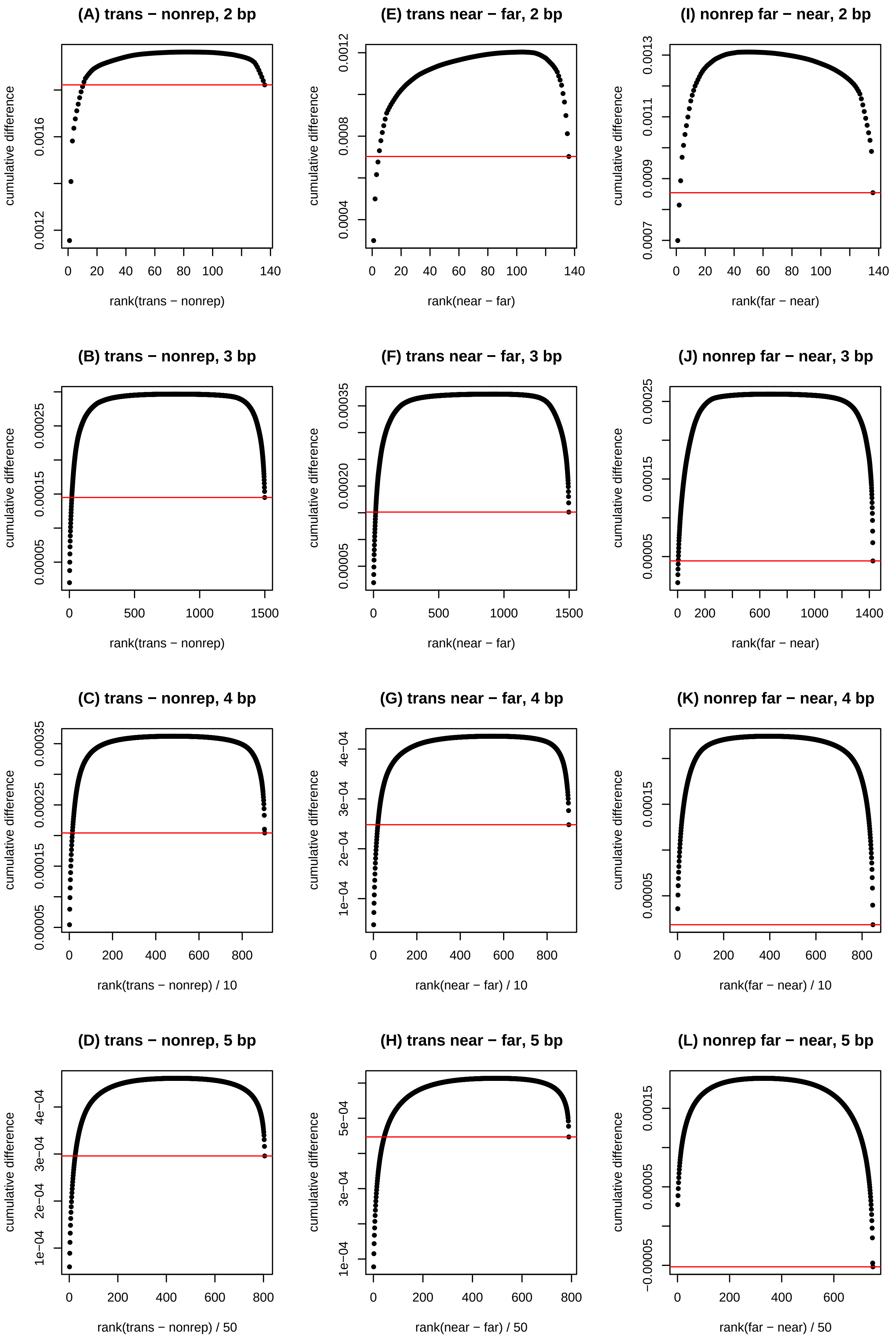
(A) trans − nonrep, 2 bp
cumulative difference
0.0012
0.0016
0 20 40 60 80 100 140
rank(trans − nonrep)
(E) trans near − far, 2 bp
cumulative difference
0.0004
0.0008
0.0012
0 20 40 60 80 100 140
rank(near − far)
(I) nonrep far − near, 2 bp
cumulative difference
0.0007
0.0009
0.0011
0.0013
0 20 40 60 80 100 140
rank(far − near)
(B) trans − nonrep, 3 bp
cumulative difference
0.00005
0.00015
0.00025
0 500 1000 1500
rank(trans − nonrep)
(F) trans near − far, 3 bp
cumulative difference
0.00005
0.00020
0.00035
0 500 1000 1500
rank(near − far)
(J) nonrep far − near, 3 bp
cumulative difference
0.00005
0.00015
0.00025
0 200 600 1000 1400
rank(far − near)
(C) trans − nonrep, 4 bp
cumulative difference
0.00005
0.00015
0.00025
0.00035
0 200 400 600 800
rank(trans − nonrep) / 10
(G) trans near − far, 4 bp
cumulative difference
1e−04
2e−04
3e−04
4e−04
0 200 400 600 800
rank(near − far) / 10
(K) nonrep far − near, 4 bp
cumulative difference
0.00005
0.00015
0 200 400 600 800
rank(far − near) / 10
(D) trans − nonrep, 5 bp
cumulative difference
1e−04
2e−04
3e−04
4e−04
0 200 400 600 800
rank(trans − nonrep) / 50
(H) trans near − far, 5 bp
cumulative difference
1e−04
3e−04
5e−04
0 200 400 600 800
rank(near − far) / 50
(L) nonrep far − near, 5 bp
cumulative difference
−0.00005
0.00005
0.00015
0 200 400 600
rank(far − near) / 50
